# Complete genome sequences of four lytic bacteriophages against multidrug-resistant *Enterococcus faecium*

**DOI:** 10.1128/mra.00688-24

**Published:** 2024-09-09

**Authors:** Oumarou Soro, Collins Kigen, Andrew Nyerere, Moses Gachoya, James Wachira, Vanessa Onyonyi, Martin Georges, Allan Wataka, Stephen Ondolo, Karen Cherono, Erick Odoyo, Lillian Musila

**Affiliations:** 1 Department of Molecular Biology and Biotechnology, Pan African University Institute for Basic Sciences, Technology, and Innovation, Nairobi, Kenya; 2 Department of Emerging Infectious Diseases, Walter Reed Army Institute of Research-Africa/Kenya Medical Research Institute, Nairobi, Kenya; 3 Department of Medical Microbiology, Jomo Kenyatta University of Agriculture and Technology, Nairobi, Kenya; Loyola University, Chicago, Illinois, USA

**Keywords:** bacteriophage, *Enterococcus faecium*, Kenya, multidrug resistance, phage therapy

## Abstract

We report the genome sequences of four *Enterococcus faecium* phages isolated from environmental wastewater in Kenya. They are double-stranded DNA phages with genomes varying in length from 42,231 to 43,348 bp, with G+C contents ranging from 34.96% to 35.2%. The genomes contain 78–82 coding sequences.

## ANNOUNCEMENT

*Enterococcus faecium* is an effective nosocomial, multidrug-resistant (MDR) pathogen, causing severe human infections (1). Bacteriophages (phages) have regained interest as alternative therapeutics to address the increase in MDR bacteria (2). We present the complete genome sequences of four *E. faecium* phages isolated in Kenya.

Phages were isolated from wastewater samples collected from the Nairobi environs, Kenya, through an enrichment method using clinical isolates of MDR *E. faecium* as hosts, as described elsewhere with slight modifications (3). Briefly, 50 mL of wastewater was centrifuged (12,000 × *g*, 10 min), and the supernatant was filtered (0.22 µm). Eight milliliters of the supernatant was mixed with 2 mL of 5× tryptic soy broth (TSB; Oxoid Ltd., Basingstoke, Hampshire, England) and 50 µL of bacterial culture grown in 1× TSB (37°C, 200 rpm, 24 h). The mixture was incubated (37°C, 200 rpm, 24 h) and clarified through centrifugation. Phages were isolated using plaque assays (4) and purified by three rounds of single plaque isolation. Pure phages were propagated on the enrichment strain in TSB and concentrated by centrifugation (10,000 × *g*, 18 h) to achieve a titer of >10^10^ PFU/mL for DNA extraction (5). Host RNA and DNA were removed using RNase A and DNase I (ThermoFisher Scientific, USA), and deproteinization was achieved by adding Proteinase K and incubating at 56°C for 1 h 30 min (3). Genomic DNA was extracted using a Norgen phage DNA isolation kit (Norgen Biotek Corp., ON, Canada), following the manufacturer’s instructions. The genomic libraries were prepared using the Illumina Collibri PCR-free ES DNA library preparation kit (Invitrogen, CA, USA) and sequenced on an Illumina MiSeq instrument with a 600-cycle MiSeq reagent kit v3, producing 350-bp maximum paired-end reads. Sequence reads were quality-controlled using FastQC v0.12.1 (6), trimmed with fastp 0.23.4 (7), and assembled with shovill 1.1.0 (https://github.com/tseemann/shovill). The quality of the assembled sequences was assessed using QUAST 5.2.0 (8). CheckV v1.0.3 (9) predicted that the genomes have a completeness of 100%, and Bandage 0.8.1 (10) was used for genome assembly visualization. Genome annotation and detection of antimicrobial resistance (AMR) and virulence genes were performed using pharokka 1.5.1 (11). Taxonomic classification was performed using Mash alignment against the INPHARED database (12). PhageLeads (13) was used to predict lifecycle and PhageTerm version 1.0.11 (14) to determine phage termini and packaging mechanisms. Nucleic acid sequence similarity searches were performed using a web-based MegaBLAST (https://blast.ncbi.nlm.nih.gov/Blast.cgi). Default parameters were used for all software tools.

The phage genomes ranged from 42,231 to 43,348 bp, with G+C contents ranging from 34.96% to 35.2%. They have linear double-stranded DNA containing 78–82 coding sequences. All four phages were unclassified at the family and genus levels. Analysis of their DNA termini identified 3′ cohesive sequences, and the BLASTn results are shown in Table 1. All the genomes lacked predicted AMR, virulence, and lysogeny genes, indicating a strictly virulent lifecycle and promising candidates for phage therapy.

**TABLE 1:** Genomic properties of the four *E. faecium* phages^*a*^

Phage name	Host strain types	Sample collection site (GPS coordinates)	BLASTn results		Genome length (bp)	G+C content (%)	No. of CDSs	Genome coverage (×)	No. of raw reads	Termini	GenBank accession no.	SRA accession no.
			% identity	Top hit								
Enterococcus phage vB_Efm3_KEN18	EFM3	Ruai Sewage treatment plant (1°14′26.6″S 37°01′01.9″E)	90.38%	Phage BUCT630 (PP434460.1)	43,348	35.16	82	68.16	231,931	Cos (3′)	PP582173	SRR28477793
			93.74%	Phage Aramis (LR990833.1)								
			93.69%	Phage dArtagnan (LR991625.1)								
Enterococcus phage vB_Efm3_KEN20	EFM3	Kahawa (1°11′58.7″S 36°55′16.9″E)	94.72%	Phage Aramis (LR990833.1)	42,434	34.96	81	69.82	463,670	Cos (3′)	PP582174	SRR28477792
			94.38%	Phage dArtagnan (LR991625.1)								
			90.75%	Phage IME-EFm1 (NC_024356.1)								
Enterococcus phage vB_Efm8_KEN21	80	Ruai Sewage treatment plant (1°14′26.6″S 37°01′01.9″E)	88.80%	Phage IME-EFm5 (NC_028826.1)	42,231	35.19	80	67.30	197,902	Cos (3′)	PP582175	SRR28477791
			88.18%	Phage IME-EFm1 (NC_024356.1)								
			87.88%	Phage Aramis (LR990833.1)								
Enterococcus phage vB_Efm10_KEN22	2672	Ruai Sewage treatment plant (1°14′26.6″S 37°01′01.9″E)	94.89%	Phage Aramis (LR990833.1)	42,487	35.2	78	69.85	110,640	Unknown	PP582176	SRR28477790
			93.74%	Phage vB_Efm_LG62 (OP018674.1)								
			93.58%	Phage dArtagnan (LR991625.1)								

^*a*^ Cos (3′), 3′ cohesive sequences; CDSs, coding sequences; SRA, Sequence Read Archive.

## Data Availability

The genome sequences of the four *E. faecium* phages are available in GenBank and Sequence Read Archive under the accession numbers listed in Table 1.
